# Unpacking medical students’ resit experiences: a qualitative study of early years medical students´ experiences of a peer-assisted learning programme during summer resit exams

**DOI:** 10.1080/10872981.2025.2477666

**Published:** 2025-03-12

**Authors:** Cate Goldwater Breheny, Angela Cebolla Sousa, Ana Vitoria Baptista

**Affiliations:** Imperial College School of Medicine, Imperial College London, London, UK

**Keywords:** Peer-assisted learning, resits, qualitative study, medical students, pre-clinical medicine

## Abstract

Resitting, being offered a ‘second chance’ at an exam following failure to achieve a passing grade, is both common and stressful in medical school. There is a significant gap in the medical education literature around evidence-based support for resitting medical students. The study explores medical student experiences of resits through a peer-assisted learning programme (PAL) delivered to early years resitting medical students at Imperial College School of Medicine (ICSM) in 2021 and 2022. To the authors’ knowledge, this is the first qualitative study analysing early years medical students’ experiences of resitting exams. The authors performed an inductive thematic analysis of 22 semi-structured interviews with early years medical students who resat exams at ICSM in 2022. The authors identified three key themes and a cross-cutting theme: Theme 1. Self: students’ individual and internal characteristics and experiences that influenced their journey of resitting exams. Subthemes included self-sufficiency and students’ emotional approach to resitting; Theme 2. Others: resitting students’ social networks. Subthemes explored students’ relationships as part of the ICSM academic community, with near-peer role models and with their emotional support networks; Theme 3. Structures: organisational and structural factors that influence student experiences of resit exams. Subthemes included academic information, welfare, and socio-economic factors. Cross-cutting theme. Stigma: experienced a lack of open communication around resitting. The data emphasises the holistic nature of resitting, with students’ self-image, their relationships with others, and the structural and institutional context all impacting on their experience, cross-cut with their experience of stigma through silence. The authors suggest that resitting is about more than academic ability: the broader context of resit stigma plays a key role in students’ experiences of resits. PAL may be a useful tool to address resit stigma alongside institutional commitments to rethink medical school culture around academic failure

## Introduction

Medical school represents formative years for students as a time of personal and professional development. It is also undeniably a period of intense pressure for many medical students [1]. Undergraduate medical students, who study medicine without a prior degree, typically joining the course at around age 18 as school leavers, may be particularly prone to finding their studies academically challenging and stressful. In the United Kingdom (UK), undergraduate medicine is the main route to becoming a doctor, in contrast to countries such as the United States of America where medicine is a postgraduate-only degree. Around a third of UK medical students struggle with their workload or their mental health, around two-thirds, experience financial difficulties, and up to 85% face a degree of a degree of burnout [2–5]. Burnout can be understood as an experience of emotional and physical depletion where students feel exhausted and disengaged [3]. These challenges are also found in other medical schools across the globe [4].

Needing to resit exams adds to these stressors, creating a particularly challenging experience for resitting medical students [2]. The idea of a resit exam is not always well defined in the literature, but can be colloquially understood as being offered a ‘second chance’ at an exam following failure to achieve a passing grade in the first exam. Resitting exams challenges students’ professional identity development, resilience, and self-belief [2]. Resitting also seems common: Holland estimates that around 10% of all medical students will resit at least once, but the authors cannot locate recent evidence for any overall numbers of resits in the U.K. [6]. In many medical schools, including Imperial College School of Medicine (ICSM), only one resit attempt at each exam is allowed before students are required to leave the medical course. Some students may instead be asked to repeat the whole academic year or apply for mitigating circumstances, official recognition that unforeseen factors such as illness or bereavement impacted their exam performance, in order to be granted a third opportunity to sit the exam. The options available to each student will typically vary depending on their individual circumstances.

Resitting students require additional academic support from faculty and peers, as well as emotional support in their personal relationships [7]. Holistic, peer-, and faculty-supported learning have all been identified as useful for resit support [6, 2, 7]. However, there is often a lack of sufficient resources, staff and financial means to provide resitting medical students with enough help, given their complex professional and personal needs [8]. This is especially the case in more remote areas, and low- and middle-income countries [9]. To the best of the authors´ knowledge, there remains a gap in the literature around both exploring in detail what medical students’ needs are during the resit period as well as an evidence-based approach around how to best support resitting students.

Peer-assisted learning (PAL) may well be one solution to the challenge of how we support resitting medical students. In PAL, ‘people from similar social groupings who are not professional teachers help others to learn and learn themselves by teaching’ [10]; in other words, students teach other students material that is covered in their degree curriculum separately to faculty-organised teaching with benefits for both student-teachers and learners. PAL has been proven to be a sustainable, feasible, and low-cost solution to support students to succeed academically [11–13]. A systematic review on peer support programs in medical school concludes that sharing near-peer experiences helps normalise difficulties encountered during medical school, such as resits [14]. PAL also positively impacts on first year medical students’ personal and professional development with senior students acting as role models [15].

However, there is still a lack of evidence around what sources of PAL support are most effective for particular student demographics and how to structure PAL programmes [14]. In the authors´ most recent search of the Web of Science database in October 2024, they observe that PAL initiatives led by medical student societies or organisations (thus not having faculty’s input nor being part of the formal medical curriculum) and relating to medical students’ preparation for exams are mostly based on practical exams. This includes anatomy spotting exams [16], and other clinical skills examinations, specifically Objective Structured Clinical Exams (OSCEs) [17], Practical Assessment of Clinical Examination Skills (PACES) [18] and the United States Medical Licensing Exams (USMLE)-style clinical exam [19]. To the authors´ knowledge, there is no published work around PAL programmes to support resitting undergraduate medical students, or around early years (usually years 1–2 of the medical degree) written, rather than practical, exams.

To address this gap in support for resitting medical students at ICSM, two authors (CGB and ACS) organised a formal peer-led Resit Support Programme through ICSM’s Medical Education Society for Phase 1a (year 1) and Phase 1b (year 2) undergraduate medical students at ICSM in summer 2021 and 2022. Following on from this, students who took part in the Resit Support Programme were interviewed to better understand their experiences. The aim of this qualitative study is to gain a deeper insight into the experiences of resitting medical students to inform future educational interventions to support students in this challenging period.

## Methods

### Context of resit exams and the PAL educational experience: Medical Education Society Resit Support Programme of 2021 and 2022 at ICSM.

After noting high rates of failure in their year group, the two first authors (ACS & CGB) decided to create a Resit Support Programme together for Phase 1a students during summer 2021. In 2022, this project was expanded to include Phase 1a and 1b students. ACS and CGB delivered the Resit Support Programme through ICSM’s Medical Education society. This is a medical student-led society at Imperial College London which aims to provide academic support and guidance to students from year 1 to final year. The society organises and creates lectures, mock exams, revision materials (such as guides to module content or flashcards), and small group tutorials for all medical students at ICSM and is well regarded by the student body.

Phase 1a ICSM students take written anatomy, bodily systems, and principles of medicine exams in May. Phase 1b ICSM students also take three written papers in May, covering lifestyle medicine and prevention, anatomy, and bodily systems, alongside a practical clinical skills exam. The principles of medicine module in Phase 1a covers pre-clinical medicine, such as immunology, biochemistry, genetics and haematology. The bodily systems module focuses on normal physiology in Phase 1a, and on pathophysiology and conditions in Phase 1b. Students also sit formative exams throughout the year alongside a summative case-based assessment module. ICSM written exams contain single best answer questions (SBAQs), very short answer questions (VSAQs), and short answer questions (SAQs). Students receive their results in July; resit examinations take place in August and each exam must be passed individually. The resit examinations, like first-sit examinations in May, examine the content of the entire module and students must pass the resit exam to continue to the next year of the course. Students only need to resit the paper they have failed. The difficulty, length, and content of the paper is similar to the May exams. There is no examination charge or fee for resit exams; however, students will need to pay an extra year of tuition should they need to resit the year, and students report incurring expenses associated with, for example, finding summer accommodation or travelling to campus to sit resit exams.

**Figure 1.**
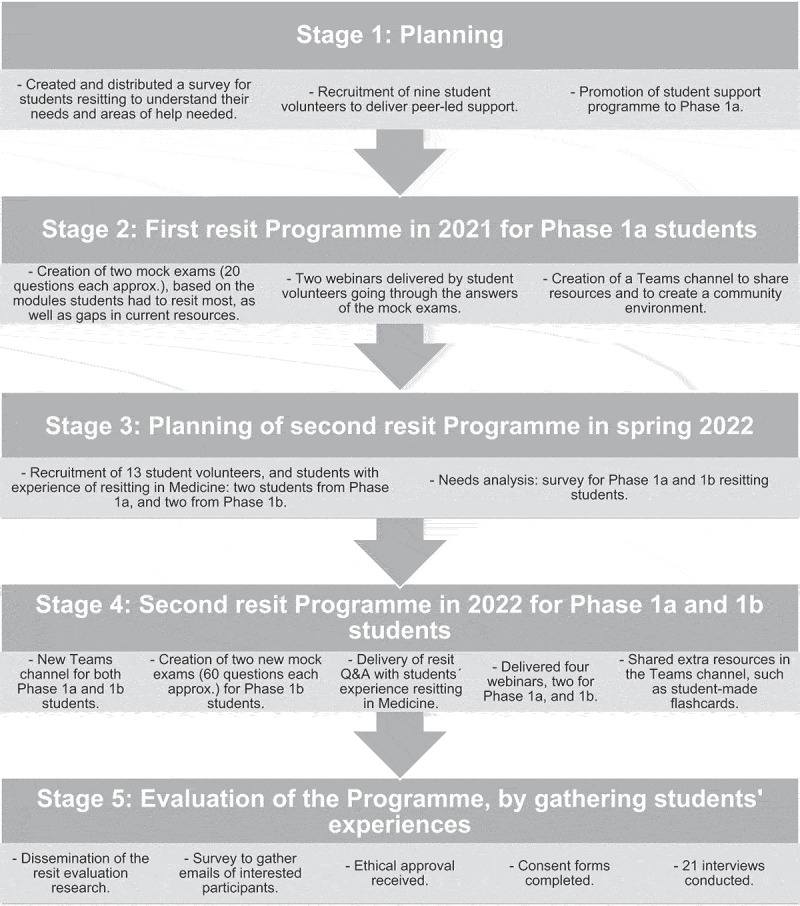
Resit support programme and research process structure diagram.

The Resit Support Programme therefore focused on supporting students with written resit exams between results release in July and resit exams in August. In 2021, the programme involved two mock exams, two webinars and a Teams channel, where more resources were shared (see Figure 1) for Phase 1a students only. In 2022, the co-first authors expanded these activities to provide support for both Phase 1a and 1b students, as detailed in Figure 1.

### Methodology

A qualitative methodology [20] was employed in this study to gain a deeper insight into the experiences of Phase 1a and Phase 1b resitting medical students in written exams in the summer 2022. The authors aimed to understand participants’ perceptions and experiences of a phenomenon (resitting written examinations) that has been understudied overall, as presented in the introduction above.

To recruit participants, the authors promoted the research study through the Resit Support Programme Microsoft Teams channel between 29 September and 1 November 2022. This Teams channel had been originally created to deliver lectures and store study materials for the Resit Support Programme. Only students who had participated in the programme were eligible to take part in the interview. Participants were offered a £10 Amazon voucher as a token of appreciation for their time. Students expressed interest in participating via an online Qualtrics form. Those who signed up were emailed a date for the interview, along with the information sheet and link to the consent form to be reviewed and signed prior to the interview. The information sheet contained information about the research project, what the interview would entail, risks and benefits to participants, and ethics considered. The full sheet can be found in the Appendix. Consent forms were collected prior to interview via Qualtrics.

Semi-structured interviews with 11 Phase 1a and 11 Phase 1b students who resat in summer 2022 and participated in the Resit Support Programme were conducted online through Microsoft Teams using secure Imperial College London accounts. These interviews were conducted over a period of 1 month in October 2022. The duration of the interviews varied according to the length of participants responses, ranging between 11 min (the shortest) and 80 min (the longest), with a median time of 26 min. The co-first authors chose to explore a range of topics across the interview and allow discussion of participants’ experience of resitting more broadly alongside their experiences of the PAL scheme. The authors´ goal was to allow participants to holistically explore their resit experience rather than simply to collect feedback on their experience of PAL. The list of prepared questions and prompts for the interviews can be found in the Appendix.

All data for this study was stored on secure, password-protected Imperial College London servers. Interview audio recordings were kept until the transcriptions were finalised, and then the recordings were deleted.

### Data analysis

Interviews were transcribed verbatim and anonymised, after which they were coded on NVivo software (QSR International Pty Ltd.; https://lumivero.com/products/nvivo/). An inductive approach to thematic analysis was followed using Braun and Clarke’s stages of thematic analysis [21].

All interview transcriptions were coded collaboratively by the first two researchers (CGB and ACS). They familiarised themselves with the dataset, formulating codes and organising them into themes, which were iteratively described and reviewed. These two researchers were the two lead coders, refining the themes and subthemes prompted by a careful analysis of the dataset together. The third researcher (AB) validated the coding process by discussing this with the lead coders and exploring the coding organisation in the NVivo file. Adding an external view to the thematic analysis process stimulated reflexive conversations and matured the interpretation that the authors present in this manuscript.

### Reflective positionality statements

CGB was a junior medical student at ICSM and peer to interviewees at the time of delivery of the Resit Support Programme and conducting project interviews. They do not have personal experience of resitting exams but were affected by the shared distress within the medical school cohort and the lack of peer-led educational support for resitting students compared to the first sit exams. They have reflected on their feelings of frustration at the experiences their fellow students have described throughout the analysis.

ACS was also a junior medical student at ICSM when she collaborated with CGB to organise the student-led programme. ACS felt particularly affected by the struggle of some of her peers during the second year of medical school, due to the difficulty of exams, insufficient holistic support for students, and by the high number of resits. ACS felt that it would be important to ensure there is more transparency in the emotional load experienced by students, to advocate for better Resit Support Programmes within medical schools, and to overall improve PAL programmes.

AB is an experienced medical educational researcher, particularly using a wide range of qualitative methodologies. As a member of staff and having not collected data for this project, AB added an external perspective to the discussion and refinement of not only the thematic analysis, but also discussion of the results.

### Ethics

Ethical approval from Imperial College London’s Faculty of Medicine Medical Education Ethics Committee (MEEC1920–181) was granted for this study.

## Results

**Figure 2.**
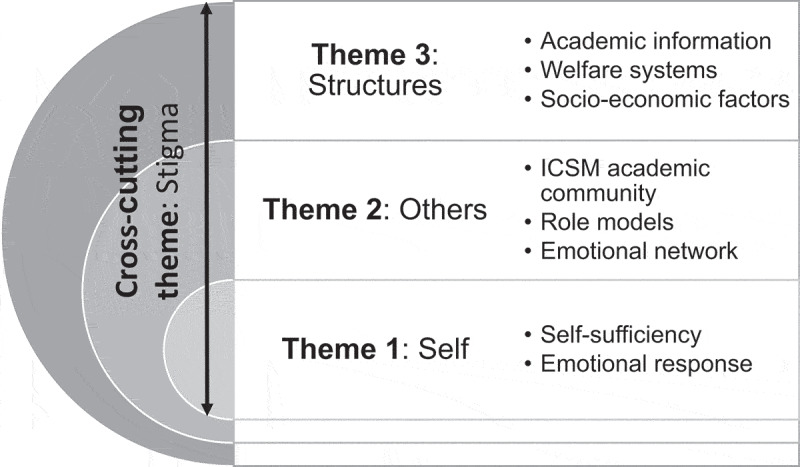
Visual representation of the thematic analysis.

When conducting reflexive thematic analysis, three key themes were identified: self, others and structures (see Figure 2).

From each theme, the team was able to identify a number of subthemes which are explored in more detail below. The themes are nested, demonstrating the dynamic interplay between factors that impact on students’ experience of resitting exams. Theme 1 explores the micro level of students’ experiences, relating to the self; theme 2, the meso-level, concerning the community; and theme 3, the macro level, encompassing structural factors. A cross-cutting theme underpinning student experiences was identified as exploring stigma, understood as a lack of open communication around the resit experience. The authors noted that stigma appeared in the data across and throughout the different themes and came to understand it as the connecting and interwoven relationship between their three themes. As such, the authors have classified stigma as a cross-cutting theme, one that recurred across each of the main three themes in their coding discussion and interlinked between the themes.

### Theme 1: self

This theme explores students’ individual and internal characteristics and experiences influencing their resit exam journey, with subthemes of self-sufficiency and students’ emotional approach to resitting.

#### Self-sufficiency

This subtheme relates to students’ internal confidence in their own individual ability to succeed academically. Resits’ impact on students’ self-perception of their own self-sufficiency was mixed. For some students, resits were an opportunity to become more self-sufficient, acting as a catalyst for them to become more confident in their study skills:

Throughout the year I realised that my learning anatomy wasn’t as good as I had wanted it to be by the end. So I think resitting the exam was good for me in terms of actually catching up the knowledge that I’d missed out in the year, or just solidifying information that I didn’t know before. – Participant 8

For these students, succeeding at resit exams was understood as within their control with changes to their studying habits:

I realised it’s happened, it’s okay, and as long as I work again I’ll probably pass, and I did. – Participant 7

However, for other students, resitting led them to perceive themselves as less self-sufficient and unable to succeed academically:

There was that feeling [resitting] would confirm that I’m not cut out for difficult [things] … If I’m not cut out for this [medicine], I don’t know what I’m cut out for because I assumed that I was capable of anything. And now suddenly I can’t do this thing [pass exams], which I’m committing all of my energy to. – Participant 14

These students often experienced resitting as an upsetting and challenging event that they did not have control over. Students could feel that they had sacrificed time and effort studying for nothing.

It was not a nice idea at all because I would feel that I wasted a whole year. All the work I put in was for nothing and that basically, all the money that I spent on this year was for nothing. That felt really bad that I would have to repeat a year and repeat a whole second year. – Participant 1

#### Emotional response

This subtheme relates to students’ personal feelings about resitting. Students differed in how they reacted emotionally to resitting, with some reporting having to ‘switch off’ emotions so they could study:

I was like, okay, head down and I’m just going to work. But it’s not healthy to have your body just switch off your emotions so that you can get ready for work. It’s not ideal. But that’s how I felt. It wasn’t good. It wasn’t nice. – Participant 12

Other students, in contrast, found resits an emotionally overwhelming process:

I think we found out on Thursday and I was actually in Barcelona when I found out. I took a day just to cry, just rest and just prepare myself. – Participant 13

Students felt sad and guilty for letting others or themselves down by resitting:

I guess you feel stupid for it, don’t you, having to resit. –Participant 10

There’s also the burden of I don’t want to disappoint my family. – Participant 4

### Theme 2: others

This theme explores resitting students’ social networks, with subthemes exploring their relationships as part of the ICSM academic community, with near-peer role models and with their emotional support networks. This theme includes and goes beyond participants’ experiences of PAL; students reflected on both formal peer assisted learning and the sense of community and emotional support social networks, often made up of immediate peers who were resitting the same exam, rather than near-peer educators.

#### ICSM academic community

Resitting students appreciated the academic community they shared at ICSM, characterised formally by student-run societies and more senior students offering academic support, as well as informally by studying together with other students.

Students felt part of a community of peer-teachers and learners who can share practical tips around study skills, which are seen as more applicable than advice from faculty:

But sometimes when students teach each other, they teach each other concepts and stuff in a more simple, easy way with mnemonics and stuff that are not exactly how faculty teaches it, I guess. – Participant 9

A lot of the time we’d do the big mind maps on the board together. Someone would write something, and the next person writes the other part, and we’d talk about it or teach each other the parts. Someone understands something, they teach it to the one that doesn’t and then vice versa. Which helps a lot more than sitting alone. – Participant 10

Sharing resources – student-authored exam papers and exam-style questions, student-authored flashcards, and notes – was a fixture in this student peer support. Students felt that this was a key part of support as they did not have access to resources from faculty, and many began their resit process by reaching out to friends and peers for resources or advice on which resources to use:

What I did for the resits was I asked my friends who passed the exams what resources they used. And they used Anki flashcards and … Basically, just Anki. I asked them to send those cards over to me. And I just powered through them as quickly as I can. And it clearly worked. *–* Participant 6

I think it was definitely the [student made] past papers [that were the most useful resource for the resits]. I think being able to practice. Because obviously learning knowledge is one thing, but then application is entirely different. So I think that was probably the best way for me to […] improve my technique. – Participant 8

The sense of community could even come from studying together in silence:

What I did with one of my friends is we would sit in silence and study together to just make sure we were locked in at our desk. And that was really helpful. – Participant 11

However, some students felt that there was less community around resits than for regular exams:

There’s all these societies [for academic support at ICSM]. It’s really, really good. But the thing is, I didn’t see that during resit period. And it felt just a bit strange. – Participant 9

#### Role models

Role models represented external figures, typically older and more experienced students, who had passed resit exams.

Students strongly valued the experiential support role models could provide:

They [peers] can tell you what to expect. Because especially it’s your first time and it’s like, I don’t know what’s going to happen, I don’t know how this works. And being able to talk to people and be like, this is how resits are going to be, this is what happens. *–* Participant 10

By existing as people who had previously passed resits, role models also acted as examples to prove that passing resits was possible and achievable for others:

Just knowing that quite a few people had to go through this. And they kept reassuring us that a lot of people actually do end up passing resits. We might think it’s a small amount, but everyone puts in the work. And there are some people who’ve had to resit all three and they managed to do that. It’s doable as long as you put in the work. Everyone kept repeating that and I thought that was good enough motivation for me to do it. ***–*** Participant 3

It was really useful seeing people had failed before, and people have got through it and that was really nice to see. – Participant 15

#### Emotional network

Students’ emotional networks represent the empathetic interpersonal relationships with others that acted as sources of support in the resit process. For many students, their emotional networks were mainly comprised of friends who were also resitting. It was important to students that they were not alone in the resit process and that they knew people like them were also resitting:

A lot of people did resit this year so it wasn’t just me by myself, so having friends that I knew who were also going through the process meant that I wasn’t lonely or anything and that we were sort of all in it together. *–* Participant 5

And I wasn’t alone even in terms of my peers because I know a few of my friends that also had to resit. I feel like I would have felt a bit differently if I was the only one I knew out of all of my friends that had to resit. I think then I would have felt a bit less supported just because I would have felt like I was the only one. – Participant 17

Additionally, students also recognised family and academic tutors as sources of emotional support:

My personal tutor was very supportive, so he was willing to meet me every week. Wanting to see where I was and stuff. So that was really helpful. – Participant 14

I felt really supported [by my family]. And they’ll be like, no, you’ll be fine for your exams. Don’t worry. And I’m Christian, so they’d always be praying for me. They’ll pray with me as well. It was really nice. – Participant 4

### Theme 3: structures

This theme relates to organisational and structural factors that influence student experiences of resit exams, with subthemes of academic information, welfare, and socio-economic factors.

#### Academic information

While students often felt well supported by individual academic staff, many felt that the academic culture and structure at ICSM created stressors around resits. Students identified a long wait for results, struggling to find information about resits, long email response times at crucial points and a lack of reassurance around next steps as important structural issues:

The least you can do is give out information about what your policies are […] Because even if you tell people you’re going to have to withdraw from the course completely, at least you feel a certainty. And actually, you can look for something else if you’re worried about that you failed. - Participant 1

I was told by my tutor and by other years that it’d be two, three weeks. But I was there waiting for four or five weeks for my results to come out. And that’s especially with the uni going to start in about a week or so. - Participant 14

There wasn’t really much else that came from [faculty] apart from when my resits were, where my resits were. And that’s about it. – Participant 2

#### Welfare systems

Students engaged with formal welfare resources such as counselling, the faculty welfare team and personal tutors. However, many received conflicting information from different welfare teams:

I went to counselling at Imperial, which was a whole other issue. But I talked to them, and she was like, oh, I’ll happily help you apply for mitigating circumstances. I’ll write a letter for you. And I was like, oh, wait, does this mean I have a chance of mitigating circumstances? So, I went to my tutor afterwards and he was like, no, you don’t. I was like, okay, fair enough. He was like, you’ve got a solid month or a month and a half before exams, there’s time. So, I was like, okay. – Participant 10

Students felt that systems were set up to be confusing and often gave delayed or unclear responses, causing them distress:

So that was one thing I wanted to review, was that how horribly they handled the mitigating circumstances. I sent them an email saying, what does mitigating circumstances actually mean? The first email response I got back was a week after resit results basically saying, yes, for the most part, so long as you have one set of mitigating circumstances in either exams, you get to resit year two. And I said that would have been great to know because I’ve had many breakdowns just over [mitigating circumstances]. – Participant 12

#### Socio-economic factors

Socio-economic factors represent structural impacts on students separate to the medical school, such as the financial costs of resits, visas, or caring responsibilities alongside summer exams.

Students referenced the financial implications of resitting, from the cost of resitting the year to losing bursary payments or struggling to find accommodation later in the year. These could be especially challenging for international students who face additional financial and immigration challenges:

Especially as an international, I’m paying a lot more, so it was quite worrying. But then I discovered the sit-out option, which is you sit out an entire year without paying school fees. But we also don’t have the student visa, which means that you can’t enter the UK. But then you can then apply for a short-term visa to come back here and take the exam. My plan was if I didn’t pass, that was the route I’m going for. – Participant 16

Students also mentioned the impact on their resit experience of caring duties and struggling to adjust to university from a disadvantaged background:

My brother just recently got diagnosed with [a health condition]. My parents don’t speak amazing English, so I was the one supporting them through all of that. I was going to a lot of meetings and stuff throughout the year. And obviously, that’s fine. I didn’t have a problem with it but it did take up a lot more time and mental energy than I thought it did. And obviously, that took a toll in terms of my education. – Participant 9

Students were often resilient and understanding about these factors, which nonetheless could seriously impact their ability to study:

I don’t have any examples of actively feeling rejected by the faculty or the union. Because I understand the thing of they don’t give you the bursary if you resit the year. And that I understand because I get that someone could just keep resitting or something. Obviously, that’s tough, it feels hard. Because it’s a lot of money to be told that you can’t have and it feels difficult because you’re like, well, I tried really hard. It wasn’t like I was just sat here. But I understand why. I don’t think that they should necessarily change that. Maybe it’d be nicer if they did a reduced bursary rather than just – because that’s going from 5000 [pounds] to go to now zero. - Participant 14

### Cross-cutting theme: stigma

Students discussed widespread stigma around resits, expressed as a lack of communication around resitting. Students felt that telling others they were resitting could expose themselves to judgement and was awkward or embarrassing:

I think it’s mainly you feel like you’ll probably get judged for it. As much as I very much know I would never judge anyone for resits, and I know most people I know wouldn’t either. But […] there will be definitely some people that are like, oh, they’re resitting the year, they’re stupid. – Participant 10

At the same, students recognised that this created a culture of silence where resits were not discussed, building a barrier to community and support:

I think there is a thing of some people are a bit, don’t want to say that they’re going to resit, I think. That they are resitting. Let’s say people who I know and I generally knew they could have told that in a chat or something, group chat, that they were resitting. And I just found out that they were resitting on the day because that’s when they’re doing their resit exam. That you don’t have … I think some people are not willing to say that they’re resitting. – Participant 1

Students who attempted to overcome this barrier and speak openly about resits found it awkward and hard to discuss:

But no one in the year group actually made it clear. It felt very sneaky because no one was saying, yes, I am resitting. And the thing is I wouldn’t mind saying individually I’m resitting. But no one else was mentioning anything so I thought everyone is acting [like] this is so embarrassing, shameful thing. – Participant 9

## Discussion

This work is, to the authors´ knowledge, the first exploration of early years medical students’ experiences of resit exams alongside a PAL programme. This data highlights the interrelationship between participants’ self-perception, their interpersonal relationships, and the institutional structures which shape their resit experience. Participants’ holistic reflections on their resit experiences reinforce recent work arguing that supporting students to succeed requires comprehensive insight into all aspects of resitting students’ medical school experience [6,2], contrasting with other discussions that focused on students’ study skills [7].

Stigma emerged as an example of a cross-cutting, holistic theme for participants. Bennion et al. [2] alongside others identify stigma as a key challenge for educators in supporting students who resit, but approach stigma primarily on an interpersonal rather than internal or structural level, and focus on direct negative experiences, such as being called stupid by teachers [2,22]. Participants here instead experienced cross-cutting stigma through silence from peers and institutions. Their experiences position resit stigma as a systemic issue, rather than one rooted in an individual’s personal academic capability or negative mindset of resitting students.

This conceptualisation of stigma aligns with an understanding of medical school structures as creating an environment where discussing resits is not psychologically safe. Psychological safety (PS) theories were originally introduced in the context of the workplace [23], but they can also be applied to medical education. In the educational context, PS can be defined as ‘students’ belief in whether it is safe for them to take interpersonal risks’ [24]. Hardie et al. [24] focuses PS on the ability to share personal and vulnerable information, such as academic failure, with others. For participants, resitting exams became unspeakable and therefore unshareable with others, creating a stigmatising and psychologically unsafe environment.

Identifying students at risk of failure and how to best support them are key concerns from educators [6, 2, 7]. This work suggests that improving psychological safety around resitting may help to support resitting medical students and avoid academic failure in general. Literature around how to create psychological safety in medical education is often limited to the classroom or clinical setting and to postgraduate doctors [25], rather than the medical school or summer resit environment. Tsuei et al [26]’s study of Canadian medical students engaged in a non-resit year-long PAL programme is a notable exception. Tsuei et al [26] explore psychological safety as an explanation for why students found the PAL scheme they describe helpful, finding that ‘a sense of P[sychological] S[afety] is strongly affected by how much the students need to continuously assess themselves against what they feel is expected of them’: psychological safety enables medical students to focus on learning rather than projecting competence at all times. Given the highly stigmatised nature of resits, this work suggests a similar process may be at work in PAL for resits, where supportive peer relationships allow students to feel confident in revision and learning rather than being afraid to share their failure to meet expectations. Participants’ experience of PAL here suggest two complimentary processes by which it may promote psychological safety around resits: both seeing near-peer students make themselves vulnerable by speaking out about having resat, and emotional support from peers. It is interesting that participants experienced feelings of psychological safety in the resit PAL scheme despite the programme lasting 2 weeks per year and not involving interpersonal mentoring. This is in contrast with Tsuei et al [26]’s PAL programme which took place over a year and where the authors argue that these longitudinal, long-term relationships with peer mentors are important to create feelings of psychological safety in medical students. The experience of psychological safety and PAL for resits may therefore be qualitatively different to that of PAL for clinical training or professional development and specific PAL programmes for resits may be useful. Equally, further research exploring differences and similarities in PAL programmes including or not including long-term mentoring may be useful to inform best practice in PAL.

Ultimately, however, addressing any systemic issue, like cross-cutting resit stigma, requires interventions which target all levels of stigma identified by participants: towards themselves, from others, and created by systems. This work suggests that PAL works well for students who have experienced academic failure. This could be expanded to create a more open culture around resits from day 1, such as by students sharing their experiences during an induction week to demystify resitting. However, any suggested PAL intervention to support resitting students must holistically acknowledge that the stigmatising environment means that students may be unwilling to speak openly about resitting: stigma acts as a barrier to challenging stigma. Any PAL intervention for resits must have clear ground rules to protect peer students who share experiences (such as requesting that information is not shared outside of PAL sessions). Additionally, they would ideally benefit from robust institutional support, including welfare and academic support, to emphasise structural openness to discussion of academic failure. The programme described here may provide guidance and inspiration for educators and medical students on implementing PAL sessions for resitting students. Enabling students to speak up about resits is key to destigmatising resit exams and then potentially preventing them.

## Strengths and limitations

To the best of the authors´ knowledge, this is the first qualitative study analysing early years medical students’ experiences of resitting exams. The recommendations and PAL programme described here are not resource or financially intensive, making these findings potentially applicable across varied different medical education contexts. The programme was volunteer-led and run without input from faculty. However, this study was conducted in a university from a high-income country with specific examination practices: new resit initiatives would have to be interpreted and adapted according to each institution’s resources and priorities.

The generalisability of these results may also be limited by their sampling methods and criteria. Participants were recruited from a group of early years medical students who had joined a Microsoft Teams channel to engage in PAL around their resits, so students who did not want to participate in PAL or who did not hear about it were excluded. They were contacted in the academic year following their resits, so students who ultimately were asked to leave medical school may have been missed, although some participants were repeating an academic year. ICSM also has a distinct approach to assessment in early years focused on high stakes end of year exams with a mix of SBAs, VSAQs and SAQs question types; the results may be less relevant to institutions with different assessment styles.

CGB and ACS developed and ran the PAL intervention from 2021 to 2022, and this study´s participants were aware of this, which may have led to bias in their responses as they did not wish to critique the programme. To mitigate this, ACS and CGB met regularly with AB, who is not a student or involved in the PAL scheme, to check the analysis and ensure it was rooted in the data. Interviewees were encouraged during the interviews to share their full perspective on the PAL scheme and were told that the co-first authors were open to critical options. Equally, interviewers’ role organising the PAL scheme may also have built trust with interviewees, who felt the authors had supported them before, enabling more in-depth reflections. The co-first authors´ involvement in the creation of the PAL intervention could have affected their interpretation of the data as well. However, the authors aimed to mitigate this by being aware of this and having regular discussions with AB.

Further research could include quantitative analysis of the PAL programmes in improving students’ grades or emotional wellbeing indicators when resitting, both of which were not in the scope of this work. Equally, further exploration of PAL schemes in other remediation contexts, such as in clinical years or for alternative examination types would deepen insight. Future mixed-methods research providing further evidence on the role of PAL programmes to support students on their subsequent exam attempts may well be useful

## Conclusions

This study explores early years medical students’ experiences of resitting exams. This data emphasises the holistic nature of resitting, with students’ self-image, their relationships with others, and the structural and institutional context all impacting on their resit journey, cross-cut with their experience of stigma through silence. Students’ experiences of resit stigma can be understood as a lack of psychological safety around resit experiences, and PAL may be a useful tool to address resit stigma.

## Appendix

- **Interview questions** [please note that the MedEd resit scheme and Teams channel are the PAL programme described in the manuscript]

- Tell us about your resit experience.
  - Prompts: which exams did you resit?
  - Had you resat exams before (if second year)? (if yes) Can you tell us more?
  - Did you have mitigating circumstances (you do not need to tell us about these in detail)?
  - Were you expecting to resit exams? If so what made you expect to do so?

How did you feel after knowing you would need to resit in August?

- Did you feel supported to take the resit exams?
  - Prompts: did you feel supported by your peers? By your peers, including your friends, your cohort, or formal societies. In which ways?/Why not?
  - Did you feel supported by faculty? In which ways?/why not
  - Did you feel supported by your family? Why or why not?
  - What, for you, would be ideal in terms of student support to take resit exams? For instance, support provided by faculty, friends, peers, student societies like meded.

- Can you tell us a little bit about how you prepared for your resit exams?
  - Prompts: did you attend the MedEd webinars? Why or why not?
  - Did you use the extra MedEd mock exams? Why or why not?
  - Did you attend the faculty resit sessions? Why or why not?
  - Did you use any other formal resit schemes, like a buddying scheme?
  - Did you reach out to peers in any way? Why or why not?
  - Did you reach out to faculty in any way? Why or why not?
  - If resitting more than one exam: what was the key difference between how you prepared for each exam?

Did you change how you studied compared to how you approached your first sit/closed book exams? (Can you tell me more?)

- What were your motivations to join the MedEd resits Teams channel?
- Did you find the MedEd resit material helpful? Why or why not?
  - Prompts: what materials did you use? Why did you choose to use these?
  - What worked well for you with the MedEd resit scheme?
  - What would you have liked to be done differently?
  - How did you feel in terms of your preparation after using the Meded resit materials?
  - If resitting more than one exam: what materials did you use for each exam?
- To what extent do you think you were disadvantaged in any way to prepare for the resit exams?
- How about taking the resit exams?
- To what extent do you think the experience of students who resit impact on the resources/opportunities available for other students resitting in the following academic years?
- What resources would you give to students who resit next year?
- What pros and cons, in your opinion, are there in resit support programmes which are designed in collaboration with students who have resat exams?
- Any other comments/suggestions?
- Thank you very much for your participation. I’ll stop the recording now?
